# Improving Oral Health After Spinal Cord Injury: A Scoping Review of Barriers, Facilitators, Current Interventions and Their Effectiveness

**DOI:** 10.1002/cre2.70310

**Published:** 2026-02-23

**Authors:** Md. Nazmul Huda, Ajesh George, Masoud Golakani, Thomas G. Elphick, Sachin Shetty, Akriti Biswas, Shilpi Ajwani

**Affiliations:** ^1^ School of Clinical Medicine, The University of New South Wales (UNSW) Sydney NSW Australia; ^2^ Australian Centre for Integration of Oral Health (ACIOH), Western Sydney University Ingham Institute for Applied Medical Research Liverpool NSW Australia; ^3^ Faculty of Health, School of Nursing & Midwifery Western Sydney University Penrith NSW Australia; ^4^ Faculty of Medicine School of Medical Sciences, UNSW Sydney NSW Australia; ^5^ Spinal Injuries Unit, Prince of Wales Hospital, UNSW Medical School Sydney NSW Australia; ^6^ Sydney Dental School, Faculty of Medicine and Health, University of Sydney, and Oral Health Promotion & Research, SLHD Oral Health Services Sydney NSW Australia

**Keywords:** clinicians, factors, interventions, spinal cord injury

## Abstract

**Objectives:**

Oral health promotion interventions promoted by non‐dental health professionals can optimize clinical outcomes and overall well‐being among people with spinal cord injury (PWSCI). Many barriers and facilitators affect non‐dental health professionals' ability to promote oral health interventions among PWSCI. This scoping review aims to map the barriers, facilitators, and current oral health promotion interventions, and to assess their effectiveness among PWSCI.

**Materials and Methods:**

Using Arksey and O'Malley's scoping review framework, a systematic search of six databases (e.g., Embase, MEDLINE, CINAHL, Web of Science, Scopus, and PubMed) and Google Scholar for published studies inEnglish was conducted. All articles published up until November 2024 were included. The PRISMA‐Scoping Reviews Checklist was applied to guide this review's structure.

**Results:**

Of the 1352 records identified, seven studies were included. Many individual and system‐level barriers (limited resources and staff, etc.) and facilitators (education and training) reported by non‐dental health professionals (nurses, occupational therapists, clinical dietitians, speech pathologists etc.) were identified. Five oral health interventions for PWSCI were reported, including oral health education and training. These interventions optimized oral health outcomes (activities of daily living, quality of life, independence in toothbrushing, reducing gingival inflammation), improved oral health practices (e.g., sustaining long‐term dental hygiene habits and improving independence in dental hygiene habits, and accessibility to geographically dispersed PWSCI) and enhanced knowledge about dental hygiene among them.

**Conclusions:**

Non‐dental health professionals can be key in promoting oral health. Although many barriers impede their ability to promote oral health, currently, there are no interventions to address these barriers and enhance their ability to optimize oral health. Therefore, more research is needed to identify appropriate interventions that can enable non‐dental health professionals to address the barriers, identify oral health issues, and integrate oral health into general healthcare.

## 1 Introduction

Spinal cord injury is a severe condition that results in neurological dysfunction and impairment below the level of the lesion, for which there is currently no effective treatment (Cristante et al. 2012). With world population growth and improved healthcare access, the number of people with spinal cord injury (PWSCI) has significantly increased in recent decades. The global prevalence rate of SCI increased by 81%, from 11.4 million in 1990 to 20.6 million in 2019 (Safdarian et al. 2023). Accordingly, appropriately managing this population's healthcare requirements is crucial to promoting positive health outcomes, including oral health. Like other disability groups, PWSCI have worse oral health compared to the general population (Pakpour et al. 2016). Poor oral hygiene in PWSCI is predominantly associated with physical disability, resulting in inadequate tooth brushing, not flossing, and using alcohol‐based mouthwash solutions (Pakpour et al. 2016; Sullivan et al. 2013). Poor oral health is also associated with respiratory complications in patients with SCI. An established link exists between increased oral bacteria, due to poor oral hygiene habits or dry mouth and aspiration pneumonia (Pace and McCullough 2010). This is particularly important since the leading cause of death for PWSCI has recently changed from renal failure to respiratory infections associated with poor oral health (DeVivo et al. 2022). Therefore, the role of non‐dental professionals in promoting effective oral health management among this highly dependent SCI community is essential, given the unique physical and health challenges PWSCI face. Non‐dental professionals (e.g., registered nurses, clinical nurse specialists, clinical nurse instructors, nursing assistants, enrolled nurses, occupational therapists, etc.) play a key role in this regard.

Various factors can facilitate oral care (OC) and the ability of non‐dental health professionals to provide OC for people with special needs (e.g., PWSCI) (Bagdesar et al. 2024). For example, education and training among non‐dental health professionals by dental professionals on the impact of SCI on oral health can facilitate the provision of OC among PWSCI (Karthikayan et al. 2018), allowing non‐dental health professionals to address oral health concerns proactively and indicating the integration of OC into primary health care (Maxey et al. 2017). Integrated multi‐disciplinary care involving dental and non‐dental health professionals can address PWSCI's physical and emotional needs (Bagdesar et al. 2024). Such integration can also optimize the management of chronic diseases, such as diabetes and cardiovascular diseases, which are often related to poor oral health (Glick and Greenberg 2017) and can reduce barriers affecting the OC of PWSCI (Bagdesar et al. 2024). This indicates the growing importance of collaboration between dental and non‐dental health professionals and the latter's role in optimizing OC among PWSCI.

Evidence also documented the barriers affecting the ability of non‐dental health professionals to promote oral health among PWSCI. Some non‐dental health professionals often feel unprepared and hesitant to provide oral care for PWSCI (Alamri 2024). Inadequate funds and a lack of specialized training may also compound the provision of adequate oral care (Barnett et al. 2015). Furthermore, poor communication between dental and non‐dental health professionals can also lead to poor oral care (Barnett et al. 2015). Another barrier might be a staff shortage, which could result in poor delivery of oral care among PWSCI (Bagdesar et al. 2024). A study found that 18% of PWSCI were unaware of their dental decay, and 60% did not know that they had periodontal disease (Sullivan 2012). PWSCI are less likely to search for oral health information online than the general population (Yuen et al. 2011). This lack of recognition of oral health potentially delays preventive treatment by non‐dental health professionals, leading to poor oral health. Non‐dental health professionals' limited knowledge and poor perception of PWSCI are other contributing factors to poor oral health in PWSCI (Yuen et al. 2010). Considering the barriers affecting non‐dental health professionals' ability to provide oral care services for PWSCI, it is crucial to enable their capacity through various strategies and interventions, given that clinicians are open to providing oral care (Bagdesar et al. 2024).

Despite the existing literature on the barriers and facilitators affecting the non‐dental professionals' ability to promote oral health among PWSCI (Bagdesar et al. 2024; Khattar 2009), there is limited synthesis of current evidence in this area. Existing reviews have primarily focused on dental professionals' perspectives on barriers and facilitators affecting preventive oral health care (Leggett et al. 2024), dental health problems of PWSCI, (e.g., gingival bleeding, plaques, dental caries, and periodontal conditions) (Alfaqeeh et al. 2020) barriers and facilitators PWSCI experience (Samuel et al. 2024) and dental care utilization factors among CALD carers, such as mothers and parents (Marcus et al. 2023). None of these reviews has addressed the barriers and facilitators influencing the capacity of non‐dental professionals to promote oral health. Consequently, reviews focusing on this area are limited.

Likewise, to our knowledge, there are no reviews on current non‐dental health professionals‐supported oral health promotion interventions and strategies among PWSCI. Although there is evidence on oral care among PWSCI (Bray et al. 2022; Coker et al. 2023), the findings of these studies did not highlight the oral health promotion interventions supported by non‐dental professionals. Moreover, the findings of the studies that included non‐dental workforce to promote oral health promotions were not systematically summarized and synthesized (Yuen 2013; Yuen and Pope 2009; Maresca et al. 2024; Nelson and Kelley 1983; Xiang et al. 2022).

Taken together, to our knowledge, there is no review of the scientific literature that has synthesized the current barriers and facilitators shaping non‐dental health professionals' ability to promote oral health among PWSCI or the interventions and strategies to improve the oral health of PWSCI. The World Health Organization (WHO) (2023) recognizes oral health as an integral part of general health and the role of multi‐disciplinary efforts involving non‐dental health professionals in oral health. Considering the gap in the literature and in alignment with the WHO framework, this scoping review aims to identify and map the barriers and facilitators influencing non‐dental health professionals' ability to promote oral health and summarize current interventions, particularly those supported by non‐dental professionals, and their effectiveness among PWSCI. This review will also help identify gaps in service delivery and patient care, and provide valuable insights that can inform practice, policy‐making, and future research needs on oral health promotion among PWSCI involving non‐dental health professionals.

## 2 Methods

This review adopted the five steps of the Arksey and O'Malley methodological framework for scoping reviews since it is commonly and most frequently cited methodological approach for scoping reviews, and it gives a clear, stepwise yet flexible structure that suits exploratory research questions (Arksey and O'Malley 2005). The PRISMA‐ScR (Preferred Reporting Items for Systematic reviews and Meta‐Analyses extension for Scoping Reviews) Checklist was applied to guide this review's structure (Tricco et al. 2018) (Supporting information S1: File 1). The PRISMA‐ScR provides a clear and systematic framework that is widely used in scoping reviews (Tricco et al. 2018). This framework is also useful as it offers structured guidance for conducting and reporting scoping reviews, improving both methodological rigor and transparency in this kind of knowledge synthesis (Tricco et al. 2018). This framework is specifically designed to map the existing evidence in scoping reviews. As we aim to explore the barriers, facilitators, and oral health promotion strategies affecting non‐dental health professionals among PWSCI, this framework is suitable for the explanatory nature of our scoping review. Given the limited literature on oral health interventions after spinal cord injury, a scoping review was appropriate for this study to map out the available literature, identify key concepts, and highlight areas for future research. The study protocol was registered in the Open Science Framework (OSF) Registry (Registration DOI: https//doi.org/10.17605/OSF.IO/WXFBH).

While a quality assessment is not a mandatory measure in a scoping review that uses the Arksey and O'Malley methodological framework for scoping reviews (Arksey and O'Malley 2005), our review undertook a quality assessment to understand the rigor of included studies to enhance up‐take and use of findings by policy makers (Grant and Booth 2009). A quality appraisal can also improve the scoping review's future research and practice recommendations in this area (Pham et al. 2014). The quality appraisal of the included articles was assessed using the Mixed Methods Appraisal Tool (MMAT, version 18) (Hong et al. 2018). The MMAT tool was validated and extensively applied to assess the quality of papers with various methods (Pace et al. 2012). The MMAT assesses the risk of bias of peer‐reviewed articles using a set of 7‐item questions. Of these, two questions were general for all empirical research designs. In contrast, five questions for each of the research designs were specific, assessing quantitative descriptive, quantitative randomized controlled trials, qualitative, and mixed methods designs differently. For each appraisal question in the MMAT, there are three potential answers which can be scored: “yes,” “I can't tell,” and “no” (Hong et al. 2018). In this review, a “yes” answer was scored 1, and an “I can't tell” or “no” answer was scored 0. The cumulative scores of assessed articles were summed up and rated on a scale of 0–7. Studies with a score of four and above were considered to have sound methodological quality (see Tables 1 and 2). Quality assessment was independently completed by M.N.H. and A.B., with any discrepancies resolved through discussion with the third reviewer (A.G.). Due to the limited number of studies we found, no article was excluded from this review based on quality, but the evaluation allows us to compare findings based on quality (Whittemore 2005) (Supporting Information S2: File 2).

**Table 1:** Descriptions of studies included in the review concerning barriers and facilitators affecting the ability of non‐dental health professionals.

Authors, publication year, titles	Country	Research methods, data analysis, sample size (*n*)	Non‐dental health professionals’ characteristics	Main findings			
				Types of non‐dental health professionals who experienced barriers and facilitators	Barriers non‐dental health professionals experienced in promoting oral health among PWSCI	Facilitators non‐dental health professionals experienced in promoting oral health among PWSCI	Quality assessment
Bagdesar, M., Samuel, R., Brown, T. D., Shetty, S., Kaur, J., Kong, A. C.,… & Ajwani, S. (2024). Integrated OC for patients with spinal cord injuries: perceptions of non‐dental professionals. Disability and Rehabilitation.	Australia	a. Qualitative descriptive design. b. Focus groups. c. Thematic analysis. d. *n* = 35	a. Mean age of the participants was 38 ± 11.3 years (range 22–62 years). b. 76% were female. c. More than half of participants worked in the rehabilitation SCI unit (65%), while the remaining worked in the acute SCI ward. d. Most (83%) participants were nursing staff, whereas the other 17% were allied health staff. e. Participants had an average of 9.3 years in current role experience (CRE) in SCI care (range 1–27 years). f. Sources of OC information and knowledge varied, with nearly a third (29%) obtaining their knowledge from personal experiences and habits.	a. Nursing staff (registered nurses, clinical nurse specialists, assistants in nursing and enrolled nurses). b. Allied health professionals (e.g., occupational therapists, speech pathologists, clinical dietitians).	**A. System‐level barriers:** ***Time constraints*** a. Limited time led to the inconsistencies in OC provision. ***Low priority*** b. OC was deemed as low priority. ***Limited access to OC*** c. Long waiting times for oral treatment in hospital settings. d. PWSCI had limited access to dental treatment. e. Staffing shortage acted as a barrier to the provision of OC. ***Lack of supportive resources*** f. A lack of structured OC assessments. g. Limited hospital dental resources in the rehabilitation SCI wards, with no OC products supplied to patients. h. SCI wards had limited OC resources and products. **B. Personal‐level barriers:** ***Limited acceptability*** i. PWSCI’ reluctance to receive OC. ***Lack of family support*** j. PWSCI did not have any family to assist in obtaining OC products.	**A. System‐level facilitators:** ***Supportive resources*** a. Adapting dental products (e.g. electric toothbrushes) to facilitate OC provision to PWSCI. b. Hospitals can offer dental products to PWSCI, including electric toothbrushes. ***New implementing protocols*** c. The implementation of oral health assessments in admission and discharge planning to prevent the area from being overlooked. d. Multi‐disciplinary meetings to discuss oral health concerns. e. Involvement of nurses and occupational therapists in oral health promotion and the admission process to facilitate independent OC. ***Education*** f. Additional oral health education and a combination of online and face‐to‐face training for clinical staff.	Methodologically sound
Khattar (2009). Oral health needs assessment of adults with spinal cord injuries resident in an institution.	India	a. Cross‐sectional design. b. Survey. c. Descriptive statistics. d. *n* = 79	a. Most staff (69; 87.3%) were aged 16–35 years. b. Seven staff were in the 35–55 age group and 3 were in the 56+age group. c. 18 (23.1%) staff were employed in the Indian Spinal Injuries Unit for less than 1 year. d. 26 (33.33%) were employed for 1–2 years. e. 22 (28%) for more than 2 and less than 5 years. f. Only 13 (16%) staff had been employed for more than 5 years.	a. Nursing and care staff.	**A. System‐level barriers:** ***Time constraints*** a. Limited time of staff (1.3%). ***Low priority*** b. Staff not interested in OC (1.3%). **B. Personal‐level barriers:** ***Limited acceptability*** c. Patients’ gagging and feeling sick was a great barrier (17.1%). d. Patients not opening mouth was another barrier (16.3%).	**A. System‐level facilitators:** ***Implementing new protocols*** a. Staff's critical role in promoting oral health (60.0%). b. Staff's involvement in cleaning of teeth or dentures of PWSCI (70.9%). ***Education*** c. Staff's further education in oral health (84.1%).	Methodologically sound

**Table 2:** Descriptions of studies concerning current oral health promotion interventions, their characteristics, and their effectiveness are included in the review.

Authors, publication year, titles	Country	Research methods, data analysis, sample size (*n*)	Characteristics of PWSCI	Main findings			
				Current oral health promotion interventions	Characteristics of current oral health promotion interventions	Intervention outcomes/effectiveness of current oral health promotion interventions	Quality assessment
Yuen and Pope (2009). Oral home telecare for adults with tetraplegia: a feasibility study.	USA	a. Questionnaire survey and in‐depth interviews. b. Analysis technique not specified. c. *n* = 2.	a. Both had SCI at C4–C7 levels and C5–C6 levels. b. Both participants had adequate motor control and were able to follow the instructions to manage the videoconferencing equipment. c. They did not depend on others to complete the OC task.	Oral home telecare training and education via video conferencing.	**Involvement of non‐dental professionals** a. Was promoted by a dental hygienist and an occupational therapist. b. In partnership with a dental hygienist, the therapist used videoconferencing to incorporate repeated training, supervise practice of oral hygiene, and give instant corrective feedback and positive reinforcement of the correct, safe, and independent use of adaptive oral hygiene devices for the participants.	**Improved oral health knowledge** a. Improving knowledge of dental hygiene. **Improved oral health practices** b. Improved independence in maintaining dental hygiene. c. Optimizing accessibility to geographically dispersed people with tetraplegia.	Methodologically sound
Yuen (2013). Effect of a home telecare program on oral health among adults with tetraplegia: a pilot study.	USA	a. Questionnaire survey and in‐depth interviews. b. Wilcoxon signed‐ranks test. Qualitative data analysis technique not specified. c. *n* = 8	a. Experienced a traumatic SCI. b. Were living in the community. c. Were able to hold a manual toothbrush with the help of a universal cuff to brush teeth. d. Had difficulty in handling a manual toothbrush to maintain oral hygiene.	Oral health telecare program.	**Training and duration** a. Individualized oral hygiene training on the applications of assistive devices through personal computer‐based videoconferencing. b. An average of five videoconferencing sessions across 3 months of oral health training on using assistive devices. c. The first two sessions lasted about 30 min, and each following session lasted about 15 min. d. Training sessions focused on supervised oral hygiene practice and providing immediate helpful feedback and positive support for using adaptive oral hygiene devices. **Involvement of non‐dental professionals** e. Was promoted by dental hygienists and occupational therapists. f. Dental hygienist conducted baseline dental assessments. g. The dental hygienist explained the various features of rechargeable, powered Oral‐B oscillating‐rotating‐pulsating toothbrushes to participants and taught them the proper way of operating toothbrushes. h. They provided participants with a brief oral hygiene instruction. i. Occupational therapists provided oral hygiene training via videoconferencing. j. Included four videoconferencing training and education sessions on motivation and proficiency in manipulating dental cleaning devices for oral hygiene performance for 4 weeks. k. Each training session lasted 20–30 min.	**Improved oral health outcomes** a. Reduced gingival inflammation and improved gingival health among PWSCI. **Improved oral health practices** b. Increased oral hygiene frequency. c. Led to sustaining long‐term dental hygiene habits.	Methodologically sound
Maresca, G., Latella, D., Formica, C., Veneziani, I., Ielo, A., Quartarone, A.,… & De Cola, M. C. (2024). The effects of home automation on personal and social autonomies in spinal cord injury patients: a pilot study.	Italy	a. Randomized controlled trial b. Mann–Whitney *U* test and Fisher's Exact Test, ANCOVA) c. *n* = 50	a. Experienced SCI. b. Attended home automation (HA) laboratory. c. Were able to follow verbal instructions.	Home automation laboratory training	**Training and duration** a. PWSCI were trained using advanced technologies available in the HA room (HA training). b. The training involved 24 sessions over a period of 8 weeks with 3 sessions per week, each lasting for approximately 60 min. c. PWSCI were trained and supported by the therapist in performing day‐to‐day tasks with HA, including oral hygiene, and more. d. Guided through exercises that involved all limbs with a focus on promoting independence in ADL. e. The HA training involved group activities with 3–5 patients per group, and the sessions took place in a dedicated HA room. f. A brush with a gentle design was used by users. **Involvement of non‐dental professionals** g. Supported by occupational therapists. h) A neurologist and a psychologist assessed patients before and after treatment. i) Occupational therapists administered treatments.	**Improved oral health outcomes** a. The effect of the experimental treatment showed an improvement in all patients test scores in the experimental group, especially regarding ADL (e.g., oral hygiene and quality of life). b. Stimulated circulation in the gums through its massaging action.	Methodologically sound
Xiang, R., Xu, F., Yin, Z., Ji, L., and Xu, Q. (2022). Effect of comprehensive nursing on traumatic paraplegia patients by evaluation of magnetic resonance imaging features.	China	a. Randomized controlled trial b. ANOVA c. *n* = 60	a. 38 males and 22 females with traumatic paraplegia due to SCI b. The average age was 35.72 ± 3.14 years old, with an age range of 20–70 years old.	Routine and comprehensive nursing on rehabilitation etc.	**Training and duration** a. Routine nursing included daily nursing and real‐time monitoring of patients’ vital signs. b. Passive and active training were performed on the affected limbs of patients with stable conditions for paraplegia and muscle atrophy of the lower limbs. c. Joint fexion training was carried out 3–4 times a day, 10 min each time. d. Patients were assisted in doing upper limb activities, such as pulling pullers and lifting dumbbells that could exercise muscle strength of the upper limbs. **Involvement of non‐dental professionals** e. Facilitated by nursing staff. f. Nursing staff explained the disease and health knowledge.	**Improved oral health outcomes** a. The scores for oral hygiene and quality of life in the experimental group were significantly higher than those in the control group 3 months and 6 months after discharge (*p* < 0.05).	Methodologically sound
Nelson and Kelley (1983). Patient and family workshops: A new teaching approach for spinal cord injury.	USA	a. Qualitative workshops with patients and caregivers b. Analysis technique not specified c. *n* = 23	a. 42 newly injured patients were admitted. b. Many patients’ families on this unit are from rural areas.	Training/teaching program to address the needs of SCI patients.	**Training and duration** a. The training workshop with a focus on independent living is held twice a month. b. Four components of the SCI training patient and family training workshop was developed for the 23 active rehabilitation patients on a 42‐bed SCI unit. c. This program included classes and individual practice sessions. d. Participants completed ADL activities to maximum capabilities, including dental hygiene. **Involvement of non‐dental professionals** e. A clinical nursing instructor coordinates the training/teaching program, documentation and follow‐up.	**Improved oral health outcomes** a. Inadequate information on clinical outcomes b. The comprehensive aspects of care have proven to be less than effective. c. A yearly evaluative study revealed the program effectively meets the needs of PWSCI.	Methodologically not sound

### 2.1 Identifying the Research Questions

As this review aims to explore current interventions supported by non‐dental health professionals in promoting oral health among PWSCI, we sought to “generate a breadth of coverage” (Arksey and O'Malley 2005) of barriers and facilitators affecting non‐dental health professionals' ability, oral health interventions and their effectiveness among PWSCI. Non‐dental health professionals include all community‐based health professionals other than dental professionals. The following two research questions guided the search strategy.

- What barriers and facilitators affect non‐dental health professionals in promoting oral health among PWSCI?
- What are the characteristics and effectiveness of current oral health promotion strategies among PWSCI?

### 2.2 Identifying Relevant Studies

To identify the studies, the Embase, MEDLINE, CINAHL, Web of Science, Scopus, and PubMed databases were searched (Supporting Information S3: File 3). The search strategy, including all identified keywords, Boolean operators, truncations and index terms, was adapted for each included database. We also searched the first few pages of Google Scholar and hand‐searched the references of published systematic reviews. This review included all articles published up to November 2024.

Articles pertaining to at least one of the focus areas were included. Articles were included if they focused on (a) barriers and enablers influencing non‐dental health professionals' ability to promote oral health, (b) oral health and SCI, (c) oral health interventions for PWSCI, and (d) outcomes such as improved activities of daily living, knowledge, and confidence of PWSCI. Articles relating to non‐dental professional‐led interventions were excluded if they (a) were not published in English, (b) did not clearly identify SCI as the study cohort, (c) did not focus specifically on oral health in PWSCI, or (d) had limited irrelevant information.

### 2.3 Study Screening and Selection

**Figure 1:**
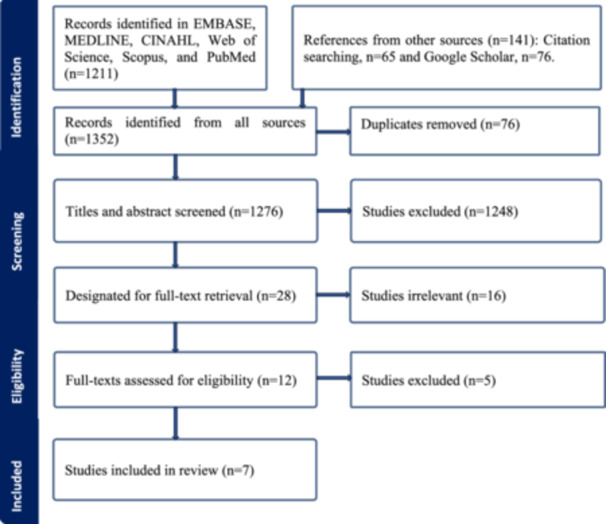
Study selection process (PRISMA‐ScR) (Tricco et al. 2018).

Figure 1 shows the study screening and selection procedure. After the search, all found studies were gathered and loaded into the Covidence systematic review software for data screening and extraction. Duplicates were eliminated. Then, two independent reviewers (M.N.H. and A.B.) screened the titles and abstracts according to the abovementioned eligibility criteria. Any conflicts and discrepancies were resolved through discussion with a third reviewer (A.G.) to reach a consensus. The exact process was subsequently repeated for full‐text screening. The reasons for exclusion were recorded.

### 2.4 Data Charting/Extraction

A data extraction form was completed for each included study (see Supporting Information S4: File 4). The following information was retrieved from the included studies: title, author, publication year, country, research design, data analysis, sample size, characteristics of PWSCI, barriers and facilitators affecting non‐dental health professionals' ability to promote oral health, types of non‐dental health professionals, current oral health promotion interventions, including their characteristics (duration of intervention, target audience, etc.), and their effectiveness interventions/intervention outcomes.

### 2.5 Collating, Summarizing, and Reporting the Results

Consistent with existing reviews (Keboa et al. 2016; Gomez‐Rossi et al. 2020), this review followed the three stages recommended by Levac et al. (2010) to generate results: (a) the first author developed a single table containing information recorded from each article; (b) four reviewers read the extracted information and did a basic descriptive analysis; and (c) then, they pooled, summarized and analyzed similar data thematically (Vaismoradi et al. 2013) according to the current review's aims, such as aim one: barriers and facilitators affecting non‐dental health professionals and aim two: characteristics and effectiveness of current oral health promotion strategies. A multi‐step mapping was conducted, with barriers mapped first, followed by facilitators. These barriers and facilitators were grouped into three main themes (individual‐level barriers, system‐level barriers, and system‐level facilitators), each with multiple subthemes, as detailed in Table 1. Regarding the second aim, a similar process was followed with two themes: (a) current oral health promotion interventions and their characteristics and (b) intervention outcomes/effectiveness of current oral health promotion interventions. Then, the characteristics of the current oral health promotion interventions were classified into two categories: involvement of non‐dental professionals and training and duration. Likewise, the outcomes/effectiveness of current oral health promotion interventions were grouped into four sub‐themes: improved clinical outcomes, improved practice, accessibility and improved knowledge. This mapping was conducted in line with the guidelines of the scoping review (Tricco et al. 2018) and existing literature (Gomez‐Rossi et al. 2020). The concepts from the current review's objectives informed the deductive codes. Two researchers (M.N.H. and A.B.) also developed emergent inductive codes independently throughout this process. The inductive and deductive codes were then merged to provide a comprehensive understanding. Coding bias and code inconsistencies were mitigated to reach consensus by holding a meeting with the team members (M.N.H., A.G., S.A., and M.H.G.).

## 3 Results

The results of this scoping review are presented in the following focused areas:

### 3.1 Search Results

In total, 1352 records were identified from seven databases, citation searching, and Google Scholar searches. After removing duplicates and screening titles, abstracts, and full texts, eight studies were identified for inclusion in this review. The reasons for excluding studies were: (1) not focused on SCI, (2) not peer‐reviewed, (3) not focused on oral health promotion interventions, (4) not focused on non‐dental health professionals, (5) not published in English, (6) full‐text unavailable, and (7) limited information on oral health interventions and their effectiveness. Search results at each stage of the review process are illustrated in Figure 1 using the PRISMA‐ScR diagram.

### 3.2 Characteristics of Included Studies Exploring Barriers and Facilitators

Table 1 summarizes the characteristics of the included studies and non‐dental health professionals. The two studies applied a qualitative descriptive design (Bagdesar et al. 2024) and cross‐sectional design (Khattar 2009), respectively. One study used thematic analysis, while another used descriptive statistics. The two studies had sample sizes of 35 and 79, respectively.

The studies involved a range of non‐dental health professionals, including occupational therapists, rehabilitation professionals, speech pathologists, clinical dietitians, registered nurses, clinical nurse specialists, clinical nurse instructors, nursing assistants and enrolled nurses. The most common professionals identified were nursing staff. Although non‐dental health professionals' ages ranged from 16 to 62 years, most non‐dental health professionals were in the 16 to 35‐year age group. The included studies had limited information on gender, income, and ethnicity.

### 3.3 Barriers Affecting Non‐Dental Health Professionals' Ability to Promote Oral Health Among PWSCI

#### 3.3.1 System‐Level Barriers

The included papers mentioned multiple system‐level barriers that impeded non‐dental health professionals' (e.g., registered nurses, clinical nurse specialists, assistants in nursing and enrolled nurses, occupational therapists, speech pathologists, etc.) ability to deliver OC among PWSCI, including limited hospital dental resources, staffing shortages, staff's limited time, limited prioritization of OC, long waiting times for patients to receive OC, and a lack of structured OC assessments (Bagdesar et al. 2024; Khattar 2009). These barriers were reflected in the following statement: “SCI wards had limited OC resources and products and limited access to dental services, with patients with SCI needing to supply their own equipment. OC was deemed as low priority by most clinicians” (Bagdesar et al. 2024).

#### 3.3.2 Personal‐Level Barriers

The main personal‐level factors were related to PWSCI's intentions to receive OC in hospital settings. These included PWSCI's reluctance to receive OC, the absence of family to assist in obtaining OC products (Bagdesar et al. 2024), their gagging and feeling of sickness (18.4%), and PWSCI's apathy in opening the mouth (17.1%) (Khattar 2009).

### 3.4 Facilitators Affecting Non‐Dental Health Professionals' Ability to Promote Oral Health Among PWSCI

#### 3.4.1 System‐Level Facilitators

The studies (Bagdesar et al. 2024; Khattar 2009) also reported on multiple system‐level factors that can facilitate non‐dental health professionals' ability to promote oral health among PWSCI. These included adapting dental products (e.g. electric toothbrushes), multi‐disciplinary meetings to discuss oral health concerns, hospitals offering dental products (e.g., electric toothbrushes) to PWSCI, brochures and online modules and a combination of oral health education and online and face‐to‐face training for clinical staff. The involvement of non‐dental health professionals in oral health promotion, oral care, oral health education, cleaning of teeth or dentures of PWSCI, and the admission process to facilitate independent oral care and the implementation of oral health assessments in admission and discharge planning can also facilitate oral health promotion among PWSCI (Bagdesar et al. 2024; Khattar 2009).

### 3.5 Characteristics of Studies Involving Oral Health Promotion Strategies

This scoping review included five studies on current oral health promotion interventions and their effectiveness/outcomes. Of the five studies, three were conducted in the United States (US) (Yuen 2013; Yuen and Pope 2009; Nelson and Kelley 1983), with the remaining two studies conducted in China (Xiang et al. 2022) and Italy (Maresca et al. 2024). All studies were published in the last 41 years, with no studies published in the previous 10 years. Two studies were randomized controlled trials (RCTs) (Maresca et al. 2024; Xiang et al. 2022), two studies applied mixed methods, including surveys and qualitative interviews (Yuen 2013; Yuen and Pope 2009) and one study utilized workshops (Nelson and Kelley 1983). The included studies utilized various statistical techniques, including descriptive statistics, the Wilcoxon signed‐rank test, Fisher's Exact Test, and ANCOVA. The mean sample size was 28.6 subjects, with a median of 23 (range = 2–60) (Table 2).

### 3.6 Current Oral Health Promotion Interventions and Their Characteristics

This review identified five oral health promotion interventions and strategies facilitated by non‐dental health professionals, and the effectiveness of interventions among PWSCI. As mentioned in Table 2, the included studies reported characteristics of interventions, including the involvement of non‐dental professionals in oral health promotion interventions, oral hygiene training, and its dose (duration and frequency). For example, the first two interventions involved oral home telecare training and education programs via video conferencing, facilitated by occupational therapists and dental hygienists (Yuen 2013; Yuen and Pope 2009). While the former intervention included four videoconferencing training sessions for 4 weeks, 20–30 min each, on motivation and proficiency in manipulating dental cleaning devices (Yuen and Pope 2009), the latter involved individualized computer‐based five‐video conferencing oral hygiene training sessions for 3 months on the applications of assistive devices (powered toothbrush and adapted flosser and/or oral irrigator) (Yuen 2013).

The findings of two RCTs involved home automation laboratory training (HALT) (Maresca et al. 2024) and routine and comprehensive nursing on rehabilitation training (RCNRT) (Xiang et al. 2022) supported by occupational therapists and nursing staff in performing day‐to‐day tasks, including oral hygiene, etc., respectively. The HALT was characterized by 24 sessions over 8 weeks, with three sessions per week, each lasting approximately 60 min. PWSCI were trained and supported by therapists to perform day‐to‐day tasks with HA, including oral hygiene and more (Maresca et al. 2024). The RCNRT was performed on the affected limbs of patients with stable conditions for paraplegia and muscle atrophy of the lower limbs. Nursing staff also carried out Joint Kexion training 3–4 times a day, for 10 min each time, and helped patients perform upper‐limb activities, such as pulling pulleys and lifting dumbbells, to strengthen upper‐limb muscles (Xiang et al. 2022).

### 3.7 Outcomes/Effectiveness of Current Oral Health Promotion Interventions

The second theme comprised three sub‐themes, which are described below.

#### 3.7.1 Improved Oral Health Outcomes

In Table 2, four studies mentioned oral health outcomes of the oral health promotion interventions for PWSCI (Yuen 2013; Maresca et al. 2024; Nelson and Kelley 1983; Xiang et al. 2022). For example, the findings of the two RCTs showed improvements in ADL, including oral hygiene and quality of life in the experimental group (Maresca et al. 2024; Xiang et al. 2022). The HALT intervention focused on promoting independence in toothbrushing, with a gentle design that stimulates circulation in the gums through its massaging action (Maresca et al. 2024). Furthermore, “the oral home telecare program and the use of adaptive devices significantly reduced gingival inflammation” (Yuen 2013). However, the oral health‐related clinical outcomes of one study were not clearly described (Nelson and Kelley 1983).

#### 3.7.2 Improved Oral Health Practices

Of the five studies, two highlighted improved oral health practices. While one study discussed that the oral health telecare program increased oral hygiene frequency and led to sustaining long‐term dental hygiene habits among adults with tetraplegia (Yuen 2013), while another study mentioned that oral home telecare training and education via video conferencing improved PWSCI's independence in maintaining dental hygiene (Yuen and Pope 2009). The current review also found that oral home telecare training and education via video conferencing optimized accessibility to geographically dispersed people with tetraplegia (Yuen and Pope 2009).

#### 3.7.3 Improved Oral Health Knowledge

Improved oral health knowledge among PWSCI was another outcome identified by this review. The findings revealed that the oral home telecare training and education via video conferencing improved PWSCI's knowledge about dental hygiene: “Oral home telecare doesn't replace dentist, but … gives you knowledge you can use” (Yuen and Pope 2009).

## 4 Discussion

Given that PWSCI are a hard‐to‐reach population group, and non‐dental health professionals can play a key role in promoting oral health interventions among PWSCI, our scoping review aimed to map the literature on barriers and facilitators influencing non‐dental health professionals' ability to promote oral health, current oral health promotion interventions and strategies, and their effectiveness among PWSCI. However, only seven studies were found. These studies were conducted in lower‐middle, upper‐middle, and high‐income countries, including India, China, Australia, Italy, and the United States of America. Not surprisingly, no studies were conducted in low‐income countries. The implication is that the barriers and facilitators influencing non‐dental health professionals' ability to optimize oral health and recent oral health promotion interventions and their effectiveness for PWSCI, particularly in resource‐constrained low‐income countries, particularly African and South American countries, remain unknown. The health authorities in these countries likely overlook improving the capacity of non‐dental health professionals to promote oral health, compared to middle‐ and high‐income countries, due to limited resources, limited availability of oral health services and competing health priorities (Susarla et al. 2022).

Our findings indicate the paucity of research in this area. The small size and inadequate information may make the evidence weak. Limited and weak evidence affect the reliability of the current study's conclusions and make it challenging to make unified recommendations on how various factors enabled non‐dental health professionals. The limited, weak evidence may also make it challenging to document specific strategies enhancing non‐dental health professionals' capacity to promote oral health among PWSCI. There are several reasons for the limited and weak evidence in these areas. First, PWSCI are a small and heterogeneous group, thus posing substantial challenges for recruitment into research and reducing the generalizability of study findings (Craven et al. 2021). Second, the integration of oral health into primary care is still in its early stages of widespread implementation, and there is limited evidence on involving non‐dental professionals in oral care among PWSCI (Patro 2024). Despite limited and weak evidence, our review is unique in that it identified gaps and assessed the quality of included studies. Thus, our review provides a clear and comprehensive depiction of existing oral health promotion interventions and their effectiveness among PWSCI. This is the first study to map barriers and facilitators affecting non‐dental health professionals' ability to promote oral health and preventive interventions and their effectiveness among PWSCI.

From our review, it is clear that non‐dental health professionals can play a vital role in promoting oral health among PWSCI. We did not come across any review on non‐dental health professionals' role in optimizing oral health among PWSCI. However, an existing review on PWSCI indicated the need of training for healthcare providers (Alfaqeeh et al. 2020) while other evidence indicated that multiple individual and system‐level barriers affect non‐dental health professionals' ability to promote oral health (Smith et al. 2024). This suggests that more research is needed to develop strategies for optimizing non‐dental health professionals' capacity to promote oral health among PWSCI. Thus, the findings of the current review strengthen the existing scholarship on barriers and facilitators affecting non‐dental health professionals' ability to promote oral health, current oral health promotion interventions and their effectiveness among PWSCI.

Our review's findings revealed multiple barriers (e.g., PWSCI's unwillingness to receive OC, staffing shortage, limited structured OC assessments, limited time of staff, low dental resources, etc.) and facilitators (e.g., involvement of non‐dental health professionals in oral health promotion, oral health education and training, hospitals offering dental products, multi‐disciplinary meetings to discuss oral health concerns, etc.) affecting non‐dental health professionals' ability to promote oral health among PWSCI. These barriers and facilitators in our review are consistent with those identified in previous reviews (Göstemeyer et al. 2019; Khan et al. 2022; Paisi et al. 2019; Slack‐Smith et al. 2017). For example, the reviews of Khan et al. (2022) and Paisi et al. (2019) indicated multiple barriers (e.g. limited oral health knowledge and awareness, difficulty in accessing dental treatment, limited care, reluctance to receive care, treatment cost, etc.) and facilitators (e.g., oral health training and services for patients, easy access to dental services). However, the previous reviews have explored barriers in the context of dental settings and among other population groups, such as people affected by mental health issues (Slack‐Smith et al. 2017), homeless people (Paisi et al. 2019), and people with special care needs (Khan et al. 2022). On the other hand, the current review highlights the importance of undertaking specific targeted interventions to address barriers and enhance non‐dental health professionals' capacity in non‐dental health settings because they are often patients' first point of contact and can substantially affect oral health outcomes (Sabbahi 2023). As such, non‐dental health professionals can play an active role in improving oral health among vulnerable populations such as PWSCI.

While this review identified facilitators affecting non‐dental health professionals' capacity to promote oral health in a non‐dental environment, they do not align with specific interventions and targeted actions to resolve the barriers impacting their ability to optimize oral health among PWSCI. Evidence indicates that appropriate and targeted interventions, such as training, resources, funding and screening tools, can enable non‐dental health professionals (e.g., GPs, nurses, etc.) to address the barriers, identify oral health issues early and provide timely interventions and preventive advice to maintain oral hygiene during routine medical visits (Barnett et al. 2015; Krausch‐Hofmann et al. 2021). For example, many non‐dental health professionals, including nurses, lack special training, guidelines and confidence in delivering oral care for PWSCI, contributing to limited oral hygiene practices (Bagdesar et al. 2024). Specific training may give non‐dental health professionals adequate knowledge and skills to identify oral health problems early, reducing dental health complications (Alfaqeeh et al. 2020). As non‐dental health professionals are integral to a multidisciplinary team providing care for PWSCI, training may help nurses better collaborate with dental and non‐dental health professionals to provide patient‐centered quality of oral health care (Samuel et al. 2024). This can lead to better referral practices and create a more holistic approach to addressing the needs and care of patients (Qin et al. 2024). This aspect is critical to integrating oral health into general healthcare settings, which aligns with the framework of the WHO's Global Strategy and Action Plan on oral health (2023–2030) (World Health Organisation 2023). Furthermore, our review suggested that there were no policies or guidelines regarding how non‐dental health professionals can be supported in promoting oral health interventions for PWSCI. The absence of guidelines indicates that non‐dental health professionals may not have clear guidance on how to conduct oral health assessments or when to refer patients to dental professionals. The Oral Health Assessment Tool is recommended for use by non‐dental health professionals; however, their use varies broadly due to limited enforcement of guidelines (The Australian Commission on Safety and Quality in Health Care 2023). Therefore, it is critical to undertake evidence‐based policies and guidelines and enforce them to enhance the capacity of non‐dental health professionals so that they can be included in oral health promotion in non‐dental settings.

Our review findings should be considered with their limitations. First, we focused on electronic literature in English, which resulted in missing relevant information published in other languages. Second, there is a likelihood that our search might have failed to capture published articles due to the search methodology, in particular, the seven databases and the search strategies. However, three researchers completed an exhaustive search for articles to minimize the potential for missing relevant articles. Our scoping reviews provide areas for further research and have clinical and public health impacts.

## 5 Conclusions

This review suggested that non‐dental health professionals can be key in promoting oral health among PWSCI. Although many individual and system‐level barriers affect non‐dental health professionals' ability to promote oral health, there are currently no interventions or targeted actions to address these barriers and enhance their ability in non‐dental settings, even though they are the first point of contact for treatment. Therefore, more research is needed to identify appropriate, targeted interventions, such as training, resources, funding, and screening tools, to enable non‐dental health professionals to address barriers and identify oral health issues. Furthermore, there was a lack of oral health promotion interventions for PWSCI, and the effectiveness of these interventions among PWSCI was limited. This indicates an urgent need for research into co‐developing effective and sustainable oral health promotion interventions that can be delivered worldwide to PWSCI. Future intervention strategies should also consider the role of non‐dental health professionals in promoting and providing oral health interventions. Together, interdisciplinary collaboration and the key role of non‐dental health professionals can advance the integration of oral health into general healthcare settings.

## Author Contributions

Ajesh George and Shilpi Ajwani conceived and designed the study and reviewed the second screening of the articles. Md. Nazmul Huda and Akriti Biswas developed the search strategy, conducted the literature search, did the initial screening assessment, completed data analysis, synthesis, and interpretations, wrote the first draft, and Md. Nazmul Huda revised the manuscript. Ajesh George, Masoud Golakani, Thomas G. Elphick, Sachin Shetty, and Shilpi Ajwani reviewed the manuscript. All authors provided input into versions of the manuscript and read and approved the final manuscript.

## Consent

The authors have nothing to report.

## Conflicts of Interest

The authors declare no conflicts of interest.

## Supporting information

File 2 Quality assessment.

Supplementary Table 1: Search Strategy (Example for CINAHL).

## Data Availability

The data supporting this study's findings are available from the corresponding author upon reasonable request.

## References

[cre270310-bib-0001] Alamri, H. 2024. “Countering the Identified Barriers to Delivered Oral Care for Children With Special Healthcare Needs: A Narrative Review.” Cureus 16, no. 8: e67561. 39310384 10.7759/cureus.67561 PMC11416710

[cre270310-bib-0002] Alfaqeeh, A. A. , M. K. Assery , and N. A. Ingle . 2020. “Oral Health Care in Patients With Spinal Cord Injury: A Systematic Review.” Annals of Medical and Health Sciences Research 10: 1122–1128.

[cre270310-bib-0003] Arksey, H. , and L. O'Malley . 2005. “Scoping Studies: Towards a Methodological Framework.” International Journal of Social Research Methodology 8, no. 1: 19–32.

[cre270310-bib-0004] Bagdesar, M. , R. Samuel , T. D. Brown , et al. 2024. “Integrated Oral Care for Patients With Spinal Cord Injuries: Perceptions of Non‐Dental Professionals.” Disability and Rehabilitation 47, no. 5: 1266–1275. 38910433 10.1080/09638288.2024.2367599

[cre270310-bib-0005] Barnett, T. , H. Hoang , J. Stuart , and L. Crocombe . 2015. “Non‐Dental Primary Care Providers' Views on Challenges in Providing Oral Health Services and Strategies to Improve Oral Health in Australian Rural and Remote Communities: A Qualitative Study.” BMJ Open 5, no. 10: 009341. 10.1136/bmjopen-2015-009341 PMC4636644 26515687

[cre270310-bib-0006] Bray, E. A. , B. Everett , A. George , Y. Salamonson , and L. M. Ramjan . 2022. “Developing a Health Care Transition Intervention With Young People With Spinal Cord Injuries: Co‐Design Approach.” JMIR Formative Research 6, no. 7: e38616. 35900814 10.2196/38616 PMC9377469

[cre270310-bib-0007] Coker, J. , M. Sevigny , N. Nguyen , R. Battaglino , and L. Morse . 2023. “Factors Associated With Regular Dental Care in People With Spinal Cord Injury: A Secondary Analysis of Data From the FRASCI Study.” Topics in Spinal Cord Injury Rehabilitation 29, no. 3: 71–79. 38076285 10.46292/sci22-00052 PMC10644853

[cre270310-bib-0008] Craven, B. C. , L. Brisbois , C. Pelletier , J. Rybkina , A. Heesters , and M. C. Verrier . 2021. “Central Recruitment: A Process for Engaging and Recruiting Individuals With Spinal Cord Injury/Disease in Research at Toronto Rehabilitation Institute.” Supplement, Journal of Spinal Cord Medicine 44, no. S1: S240–S249. 34779741 10.1080/10790268.2021.1970898 PMC8604526

[cre270310-bib-0009] Cristante, A. F. , T. E. P. de Barros Filho , R. M. Marcon , O. B. Letaif , and I. D. da Rocha . 2012. “Therapeutic Approaches for Spinal Cord Injury.” Clinics 67: 1219–1224. 23070351 10.6061/clinics/2012(10)16 PMC3460027

[cre270310-bib-0010] DeVivo, M. J. , Y. Chen , and H. Wen . 2022. “Cause of Death Trends Among Persons With Spinal Cord Injury in the United States: 1960‐2017.” Archives of Physical Medicine and Rehabilitation 103, no. 4: 634–641. 34800477 10.1016/j.apmr.2021.09.019

[cre270310-bib-0011] Glick, M. , and B. L. Greenberg . 2017. “The Role of Oral Health Care Professionals in Providing Medical Services.” Journal of Dental Education 81, no. 8: eS180–eS185. 28765470 10.21815/JDE.017.025

[cre270310-bib-0012] Gomez‐Rossi, J. , K. Hertrampf , J. Abraham , et al. 2020. “Interventions to Improve Oral Health of Older People: A Scoping Review.” Journal of Dentistry 101: 103451. 32810577 10.1016/j.jdent.2020.103451

[cre270310-bib-0013] Göstemeyer, G. , S. R. Baker , and F. Schwendicke . 2019. “Barriers and Facilitators for Provision of Oral Health Care in Dependent Older People: A Systematic Review.” Clinical Oral Investigations 23: 979–993. 30707299 10.1007/s00784-019-02812-4

[cre270310-bib-0014] Grant, M. J. , and A. Booth . 2009. “A Typology of Reviews: An Analysis of 14 Review Types and Associated Methodologies.” Health Information and Libraries Journal 26, no. 2: 91–108. 19490148 10.1111/j.1471-1842.2009.00848.x

[cre270310-bib-0015] Hong, Q. N. , S. Fàbregues , G. Bartlett , et al. 2018. “The Mixed Methods Appraisal Tool (MMAT) Version 2018 for Information Professionals and Researchers.” Education for Information 34, no. 4: 285–291.

[cre270310-bib-0016] Karthikayan, R. , A. Sukumaran , K. Iyer , and M. K. Diwakar . 2018. “Spinal Cord Injury and Oral Health Status: A Systematic Review.” International Journal of Community Dentistry 6: 21–26.

[cre270310-bib-0017] Keboa, M. T. , N. Hiles , and M. E. Macdonald . 2016. “The Oral Health of Refugees and Asylum Seekers: A Scoping Review.” Globalization and Health 12: 59. 27717391 10.1186/s12992-016-0200-x PMC5055656

[cre270310-bib-0018] Khan, A. J. , B. A. Md Sabri , and M. S. Ahmad . 2022. “Factors Affecting Provision of Oral Health Care for People With Special Health Care Needs: A Systematic Review.” Saudi Dental Journal 34, no. 7: 527–537. 36267535 10.1016/j.sdentj.2022.08.008 PMC9577340

[cre270310-bib-0019] Khattar, V. 2009. “Oral Health Needs Assessment of Adults With Spinal Cord Injuries Resident in an Institution.” Journal of Indian Association of Public Health Dentistry 7, no. 13: 30–36.

[cre270310-bib-0020] Krausch‐Hofmann, S. , T. D. Tran , B. Janssens , et al. 2021. “Assessment of Oral Health in Older Adults by Non‐Dental Professional Caregivers—Development and Validation of a Photograph‐Supported Oral Health–Related Section for the interRAI Suite of Instruments.” Clinical Oral Investigations 25: 3475–3486. 33196870 10.1007/s00784-020-03669-8 PMC8137625

[cre270310-bib-0021] Leggett, H. , K. Vinall‐Collier , J. Csikar , J. Owen , S. Edwebi , and G. V. A. Douglas . 2024. “A Scoping Review of Dental Practitioners' Perspectives on Perceived Barriers and Facilitators to Preventive Oral Health Care in General Dental Practice.” BMC Oral Health 24, no. 1: 249. 38368349 10.1186/s12903-024-04022-1 PMC10874524

[cre270310-bib-0022] Levac, D. , H. Colquhoun , and K. K. O'Brien . 2010. “Scoping Studies: Advancing the Methodology.” Implementation Science 5: 69. 20854677 10.1186/1748-5908-5-69 PMC2954944

[cre270310-bib-0023] Marcus, K. , M. Balasubramanian , S. Short , and W. Sohn . 2023. “Barriers and Facilitators to Dental Care Among Culturally and Linguistically Diverse Carers: A Mixed‐Methods Systematic Review.” Community Dentistry and Oral Epidemiology 51, no. 2: 327–344. 35342972 10.1111/cdoe.12745

[cre270310-bib-0024] Maresca, G. , D. Latella , C. Formica , et al. 2024. “The Effects of Home Automation on Personal and Social Autonomies in Spinal Cord Injury Patients: A Pilot Study.” Journal of Clinical Medicine 13, no. 5: 1275. 38592129 10.3390/jcm13051275 PMC10932432

[cre270310-bib-0025] Maxey, H. L. , C. Farrell , and A. Gwozdek . 2017. “Exploring Current and Future Roles of Non‐Dental Professionals: Implications for Dental Hygiene Education.” Journal of Dental Education 81, no. 9: eS53–eS58. 28864804 10.21815/JDE.017.033

[cre270310-bib-0027] Nelson, A. L. , and B. Kelley . 1983. “Patient and Family Workshops: A New Teaching Approach for Spinal Cord Injury.” Rehabilitation Nursing 8, no. 6: 13–16. 6558752 10.1002/j.2048-7940.1983.tb02477.x

[cre270310-bib-0028] Pace, C. C. , and G. H. McCullough . 2010. “The Association Between Oral Microorgansims and Aspiration Pneumonia in the Institutionalized Elderly: Review and Recommendations.” Dysphagia 25: 307–322. 20824288 10.1007/s00455-010-9298-9

[cre270310-bib-0029] Pace, R. , P. Pluye , G. Bartlett , et al. 2012. “Testing the Reliability and Efficiency of the Pilot Mixed Methods Appraisal Tool (MMAT) for Systematic Mixed Studies Review.” International Journal of Nursing Studies 49, no. 1: 47–53. 21835406 10.1016/j.ijnurstu.2011.07.002

[cre270310-bib-0030] Paisi, M. , E. Kay , A. Plessas , et al. 2019. “Barriers and Enablers to Accessing Dental Services for People Experiencing Homelessness: A Systematic Review.” Community Dentistry and Oral Epidemiology 47, no. 2: 103–111. 30614026 10.1111/cdoe.12444

[cre270310-bib-0031] Pakpour, A. H. , S. Kumar , J. F. M. Scheerman , C.‐Y. Lin , B. Fridlund , and H. Jansson . 2016. “Oral Health‐Related Quality of Life in Iranian Patients With Spinal Cord Injury: A Case–Control Study.” Injury 47, no. 6: 1345–1352. 27085836 10.1016/j.injury.2016.03.022

[cre270310-bib-0032] Patro, S. K. 2024. “Integration of Oral Health Care Within the Healthcare System.” Frontiers in Oral Health 5: 1387141. 38562402 10.3389/froh.2024.1387141 PMC10982479

[cre270310-bib-0033] Pham, M. T. , A. Rajić , J. D. Greig , J. M. Sargeant , A. Papadopoulos , and S. A. McEwen . 2014. “A Scoping Review of Scoping Reviews: Advancing the Approach and Enhancing the Consistency.” Research Synthesis Methods 5, no. 4: 371–385. 26052958 10.1002/jrsm.1123 PMC4491356

[cre270310-bib-0034] Qin, W. , N. Liu , Q. Wang , Y. Dong , and L. Jiang . 2024. “Oral Health Literacy and Patient Education Practices Among Non‐Dental Professionals in Chongqing, China: A Cross‐Sectional Study.” Medical Science Monitor 30: e945207. 39473050 10.12659/MSM.945207 PMC11533718

[cre270310-bib-0035] Sabbahi, D. 2023. “The Effectiveness of Oral Health Education Among Non‐Dental Healthcare Professionals in Jeddah, Saudi Arabia: A Quasi‐Experimental Study.” Cureus 15, no. 11: e49187. 38130573 10.7759/cureus.49187 PMC10734712

[cre270310-bib-0036] Safdarian, M. , E. Trinka , V. Rahimi‐Movaghar , et al. 2023. “Global, Regional, and National Burden of Spinal Cord Injury, 1990–2019: A Systematic Analysis for the Global Burden of Disease Study 2019.” Lancet Neurology 22, no. 11: 1026–1047. 37863591 10.1016/S1474-4422(23)00287-9 PMC10584692

[cre270310-bib-0037] Samuel, R. , M. Bagdesar , T. D. G. Brown , et al. 2024. “Perceptions of Patients Towards Oral Health Care in a Spinal Cord Injury Rehabilitation Unit: A Qualitative Study.” Journal of Oral Rehabilitation 51, no. 9: 1701–1711. 38886619 10.1111/joor.13762

[cre270310-bib-0039] Slack‐Smith, L. , L. Hearn , C. Scrine , and A. Durey . 2017. “Barriers and Enablers for Oral Health Care for People Affected by Mental Health Disorders.” Australian Dental Journal 62, no. 1: 6–13. 27164018 10.1111/adj.12429

[cre270310-bib-0040] Smith, M. B. , E. Hitchings , and L. McBain . 2024. “Nurses' and General Practitioners' Perspectives on Oral Health in Primary Care: A Qualitative Study.” Journal of Primary Health Care 17, no. 1: 10–16. 10.1071/HC23153 40152948

[cre270310-bib-0041] Sullivan . 2012. “Perception of Oral Status as a Barrier to Oral Care for People With Spinal Cord Injuries.” American Dental Hygienists' Association 86, no. 2: 111–119. 22584448

[cre270310-bib-0042] Sullivan, A. L. , J. H. Bailey , and D. S. Stokic . 2013. “Predictors of Oral Health After Spinal Cord Injury.” Spinal Cord 51, no. 4: 300–305. 23295469 10.1038/sc.2012.167

[cre270310-bib-0043] Susarla, S. M. , M. Trimble , and K. Sokal‐Gutierrez . 2022. “Cross‐Sectional Analysis of Oral Healthcare vs. General Healthcare Utilization in Five Low‐and Middle‐Income Countries.” Frontiers in Oral Health 3: 911110. 35815119 10.3389/froh.2022.911110 PMC9259954

[cre270310-bib-0044] The Australian Commission on Safety and Quality in Health Care. 2023 . “Oral Health Care for Adult Inpatients: Recommendations.”

[cre270310-bib-0045] Tricco, A. C. , E. Lillie , W. Zarin , et al. 2018. “PRISMA Extension for Scoping Reviews (PRISMA‐ScR): Checklist and Explanation.” Annals of Internal Medicine 169, no. 7: 467–473. 30178033 10.7326/M18-0850

[cre270310-bib-0046] Vaismoradi, M. , H. Turunen , and T. Bondas . 2013. “Content Analysis and Thematic Analysis: Implications for Conducting a Qualitative Descriptive Study.” Nursing & Health Sciences 15, no. 3: 398–405. 23480423 10.1111/nhs.12048

[cre270310-bib-0047] Whittemore, R. 2005. “Combining Evidence in Nursing Research: Methods and Implications.” Nursing Research 54, no. 1: 56–62. 15695940 10.1097/00006199-200501000-00008

[cre270310-bib-0048] World Health Organisation. 2023 . “Global Strategy and Action Plan on Oral Health 2023–2030.” https://www.who.int/publications/i/item/9789240090538.

[cre270310-bib-0049] Xiang, R. , F. Xu , Z. Yin , L. Ji , and Q. Xu . 2022. “Effect of Comprehensive Nursing on Traumatic Paraplegia Patients by Evaluation of Magnetic Resonance Imaging Features.” Contrast Media & Molecular Imaging 2022, no. 1: 4712797. 36105446 10.1155/2022/4712797 PMC9444435

[cre270310-bib-0050] Yuen, H. K. 2013. “Effect of a Home Telecare Program on Oral Health Among Adults With Tetraplegia: A Pilot Study.” Spinal Cord 51, no. 6: 477–481. 23318557 10.1038/sc.2012.176 PMC3628960

[cre270310-bib-0051] Yuen, H. K. , A. Azuero , and S. London . 2011. “Association Between Seeking Oral Health Information Online and Knowledge in Adults With Spinal Cord Injury: A Pilot Study.” Journal of Spinal Cord Medicine 34, no. 4: 423–431. 21903017 10.1179/2045772311Y.0000000020 PMC3152815

[cre270310-bib-0052] Yuen, H. K. , and C. Pope . 2009. “Oral Home Telecare for Adults With Tetraplegia: A Feasibility Study.” Special Care in Dentistry 29, no. 5: 204–209. 19740151 10.1111/j.1754-4505.2009.00094.x

[cre270310-bib-0053] Yuen, H. K. , B. J. Wolf , D. Bandyopadhyay , K. M. Magruder , A. W. Selassie , and C. F. Salinas . 2010. “Factors That Limit Access to Dental Care for Adults With Spinal Cord Injury.” Special Care in Dentistry 30, no. 4: 151–156. 20618781 10.1111/j.1754-4505.2010.00146.x PMC2904989

