# Supplementary material for: Improving Oral Health After Spinal Cord Injury: A Scoping Review of Barriers, Facilitators, Current Interventions and Their Effectiveness
Source: Clin Exp Dent Res. 2026 Feb 23;12(2):e70310. doi: 10.1002/cre2.70310 (PMC12928099; doi:10.1002/cre2.70310)
Supplement: Supplementary file 1 — File 2 Quality assessment. [file CRE2-12-e70310-s002.docx]

| **Author and Year** | **Screening questions for all types** | | **1. Quantitative**  **descriptive** | | | | | **2. Quantitative**  **Randomized controlled trials** | | | | | **3. Qualitative** | | | | | **4. Mixed methods** | | | | | **Overall Score** | **Status** |
| --- | --- | --- | --- | --- | --- | --- | --- | --- | --- | --- | --- | --- | --- | --- | --- | --- | --- | --- | --- | --- | --- | --- | --- | --- |
|  | S1. Are there clear research question? | S2. Do the collected data allow to address the research questions? | 1.1. Is the sampling strategy relevant to address the research question? | 1.2. Is the sample representative of the target population? | 1.3. Are the measurements appropriate? | 1.4. Is the risk of nonresponse bias low? | 1.5. Is the statistical analysis appropriate to answer the research question? | 2.1. Is randomization appropriately performed? | 2.2. Are the groups comparable at baseline? | 2.3. Are there complete outcome data? | 2.4. Are outcome assessors blinded to the intervention provided? | 2.5. Did the participants adhere to the assigned intervention? | 3.1 Is the qualitative approach appropriate to answer the research questions? | 3.2. Are the qualitative data collection methods adequate to address the research question? | 3.3. Are the findings adequately derived from the data? | 3.4. Is the interpretation of results sufficiently substantiated by data? | 3.5. Is there coherence between qualitative data sources, collection, analysis and interpretation? | 4.1. Is there an adequate rationale for using a mixed methods design to address the research question? | 4.2. Are the different components of the study effectively integrated to answer the research question? | 4.3. Are the outputs of the integration of qualitative and quantitative components adequately interpreted? | 4.4. Are divergences and inconsistencies between quantitative and qualitative results adequately addressed? | 4.5. Do the different components of the study adhere to the quality criteria of each tradition of the methods involved? |  |  |
| Khattar (2009) | 1 | 0 | 0 | 0 | 1 | 0 | 1 |  |  |  |  |  |  |  |  |  |  |  |  |  |  |  | 4 | MS |
| Maresca et al. (2024) | 1 | 1 |  |  |  |  |  | 1 | 1 | 1 | 0 | 1 |  |  |  |  |  |  |  |  |  |  | 6 | MS |
| Xiang et al. (2002) | 1 | 1 |  |  |  |  |  | 1 | 1 | 1 | 0 | 1 |  |  |  |  |  |  |  |  |  |  | 6 | MS |
| Bagdesar et al. (2024) | 1 | 1 |  |  |  |  |  |  |  |  |  |  | 1 | 1 | 1 | 1 | 1 |  |  |  |  |  | 7 | MS |
| Nelson & Kelley (1983) | 1 | 0 |  |  |  |  |  |  |  |  |  |  | 1 | 0 | 1 | 0 | 0 |  |  |  |  |  | 3 | Not MS |
| Yuen & Pope (2009) | 1 | 1 |  |  |  |  |  |  |  |  |  |  |  |  |  |  |  | 0 | 1 | 1 | 0 | 0 | 4 | MS |
| Yuen, (2013). | 1 | 1 |  |  |  |  |  |  |  |  |  |  |  |  |  |  |  | 0 | 1 | 1 | 0 | 1 | 5 | MS |

Notes: Yes – 1.0 point; I can't tell (ICT) – 0 point; No – 0 point; MS – Methodologically Sound; blank cells – Not relevant to study
