# Supplementary material for: Improving Oral Health After Spinal Cord Injury: A Scoping Review of Barriers, Facilitators, Current Interventions and Their Effectiveness
Source: Clin Exp Dent Res. 2026 Feb 23;12(2):e70310. doi: 10.1002/cre2.70310 (PMC12928099; doi:10.1002/cre2.70310)
Supplement: Supplementary file 2 — Supplementary Table 1: Search Strategy (Example for CINAHL). [file CRE2-12-e70310-s001.docx]

# **Supplementary Table 1: Search Strategy (Example for CINAHL)**

| Item | Search strategy syntax |
| --- | --- |
| #1 barriers/facilitators | TI (knowledge OR oral health knowledge OR dental health knowledge OR practice* OR perception OR attitude* OR view* OR training OR eduction OR awareness OR factor* OR facilitator* OR enabler* OR barrier* OR practi* OR strateg* OR difficult* OR obstacle* OR limitation* OR obstacle* OR impediment* OR hurdle* OR drawback*) |
| #2 (spinal cord injury) | TI (spinal cord injury OR SCI OR spinal cord trauma OR spinal trauma OR spinal injury OR spinal injuries OR spinal cord lesion OR spinal contusion OR spinal cord contusion OR spinal compression OR spinal cord compression OR spinal distraction OR spinal cord distraction OR spinal dislocation OR spinal cord dislocation OR spinal transection OR spinal cord transection OR upper limb paralysis OR paraplegic patient* OR upper limb paralysed patient* upper limb paralysed people OR SCI patient* OR people with SCI OR paraplegia OR tetraplegia OR quadriplegia OR quadriplegic OR systemic sclerosis OR neuromuscular disabled participant*) |
| #3 (oral health) | T1 (oral health OR dental health OR oral hygiene OR dental hygiene OR dental health program* OR oral health program* OR oral health promotion OR dental health promotion) |
| #4 (Non-dental health professionals) | TI (non-dental health professional* OR spinal cord injury clinician* OR primary healthcare worker* OR integrated care OR speech pathologist* OR allied health professional* OR allied health staff OR medical specialist* OR clinician* OR staff OR rehabilitation specialist* OR occupational therapist* OR general health professional* OR general health practitioner* OR general practitioner* OR doctor* OR physician* OR nurse* OR frontline health worker* OR community health worker* OR carer*) |
| #5 | #1 AND #2 AND #3 AND/OR #4, Limiter – Publication Year: Upto Nov 2024 |
